# Tomato Urban Gardening Supported by an IoT-Based System: A Latin American Experience Report on Technology Adoption

**DOI:** 10.3390/s24237620

**Published:** 2024-11-28

**Authors:** Manuel J. Ibarra-Cabrera, Irwin Estrada Torres, Mario Aquino Cruz, Ronald A. Rentería Ayquipa, Sergio F. Ochoa, Juan Manuel Ochoa

**Affiliations:** 1 Informatics and Systems Academic Department, Universidad Nacional Micaela Bastidas de Apurímac, Abancay 03001, Peru; 111156@unamba.edu.pe (I.E.T.); maquino@unamba.edu.pe (M.A.C.); rrenteria@unamba.edu.pe (R.A.R.A.); 2 Department of Computer Science, Universidad de Chile, Santiago 8370456, Chile; 3 Office for Latin America and Caribbean, FAO—United Nations, Santiago 7630412, Chile; juan.ochoa@fao.org

**Keywords:** urban gardening, voluntary adoption of technology, Latin American experience report, hydroponic cultivation, monitoring and control system, tomato growing, gardening at family scale

## Abstract

When urban agriculture is addressed at a family scale, known as urban gardening, it is assumed as a non-commercial activity where some family members voluntarily take care of the plantation during their free time. If technology is going to be used to support such a process, then the solutions should consider the particularities of these gardeners (e.g., life dynamics and culture) to make them adoptable. The literature reports several urban agriculture experiences in Western countries and Southeast Asia; however, this activity has been poorly explored in South American countries, particularly at a family scale and considering the culture and the affordability of the solutions. This article presents an experience report of urban gardening in Peru, where a prototype of an IoT system and a mobile application were conceived, implemented, and used to support the gardening of vegetables at a family scale, considering the cultural aspects of the gardeners. This experience obtained positive results in terms of tomato production, mainly showing the system’s capability to self-adapt its behavior to consider the cultivation conditions of these urban gardeners. To the best of our knowledge, this is the first IoT system that can be iteratively adjust its behavior to improve the chances of being adopted by a particular end-user population (i.e., gardeners).

## 1. Introduction

Urban agriculture may play a role in helping to address urban food insecurity worldwide [1], and particularly in developing countries, where poverty and population tend to be concentrated in urban areas [2]. In Latin America, the rapid urban transformations of the cities and the influence of culture make urban agriculture particularly relevant [3,4]. 

This activity is frequently performed with non-commercial (gardening) or commercial (farming) purposes, often in unconventional spaces, like rooftops, backyards, or community gardens [5]. Gardening, in particular, is typically conducted on a small scale (e.g., family scale) and the activities are usually conducted during the free time of the gardeners [6]. Hydroponic cultivation and the use of greenhouses have proven to be a highly effective combination for growing vegetables at a small scale. 

In recent decades, urban gardening using hydroponics has grown considerably worldwide, influenced by the use of IoT-based systems that reduce the effort required by gardeners to grow vegetables. These systems monitor and control key variables in both the greenhouse and the cultivation infrastructure (e.g., the temperature, humidity, pH, and electrical conductivity of the cultivation substrate), by taking autonomous actions and raising awareness for the gardener [7,8,9]. 

The adoption of this supporting technology has been quite successful in many Western and Asian countries [10,11]. However, in Latin America, it has been advancing slowly. This limitation seems to be rooted in the suitability of current systems for use by urban gardeners in this region [12,13]. In support of it, several widely accepted models like TAM (Technology Acceptance Model) [14], IDT (Innovation Diffusion Theory) [15] and TPB (Theory of Planned Behavior) [16] demonstrate how technology adoption depends on the end-users’ characteristics and needs.

Although many researchers have shown that urban cultivation in this region exhibits unique cultural and social characteristics [3,17,18], little has been done to understand how these aspects can be used to inform the design of urban gardening technology for this target population. Only a few of the current IoT-based systems have been developed to support the particularities of growing vegetables in Latin American cities, considering hydroponic production at a family scale [13,19,20], but these solutions are not capable of dynamically adjusting their behavior if the gardeners change their cultivation needs or preferences.

This article presents an IoT-based system that has a configurable behavior. Thus, it aims to address the specific needs of individual gardeners, enhance its perceived value for end-users, and facilitate adoption. The system is composed of three main components: a monitoring and control system, a software backend (controller), and a mobile/web application for the gardener. 

This system was utilized to cultivate tomatoes using hydroponics at a family scale in a greenhouse located in the downtown of Abancay city, Peru. During the process, the behavior of the system was adjusted iteratively several times to address particular needs of the gardener, and thus improve the perceived value and adoptability. 

The report of this experience shows a successful experiment in terms of tomato production, but it mainly presents a strategy that enhances the system’s likelihood of adoption, by enabling self-adaptation to the gardener’s cultivation context. The previous works do not include mechanisms to flexibilize the system behavior with this purpose. Moreover, the ability to configure system behavior serves as a valuable tool for crop-science research, because it allows researchers to perform rapid prototyping of the system behavior at low cost and effort. This aspect is not addressed by the previous works.

The next section explains various cultural and social aspects that affect technology adoption by Latin American urban gardeners. Moreover, it briefly describes the cultivation of vegetables in greenhouses using hydroponics, which is the production technique considered in this experience report. Section 3 presents the related work and discusses various IoT systems that support urban cultivation scenarios. Section 4 and Section 5 detail the design and implementation of the solution, respectively. Section 6 outlines the hydroponic cultivation experience used to evaluate the support system and presents the findings. Section 7 revisits the features of the proposed system considering the related work. Section 8 discusses the contributions of this paper. Section 9 presents the conclusions and future work.

## 2. Background

In this section, we present the economic, cultural, and social factors reported in the literature that influence the adoption of urban gardening technology in Latin American contexts. We then briefly introduce hydroponic cultivation in urban greenhouses, the gardening strategy used in this experience report, which forms the context of this study.

### 2.1. Economic, Cultural, and Social Aspects Affecting Urban Gardening in Latin America 

The feasibility of adopting a certain technology depends on the cultural context in which it operates [12,14]. Therefore, designing technology to support urban agriculture in Latin American cities requires consideration of the gardeners’ cultural, economic, and social aspects, as well as the purpose of the activity and its scale. Next, we present several factors reported in the literature that influence gardeners’ willingness to adopt a specific support technology. 

*The cultural and social characteristics of urban gardeners in Latin America*. In Latin America, many urban gardeners have demanding work schedules that keep them away from home for much of the day. Upon returning, their priorities often shift to family care and leisure activities. This pattern is particularly common among urban gardeners who must balance multiple responsibilities, such as childcare or elderly care, alongside vegetable cultivation [3,18]. In these cases, the time available for gardening is often fragmented; agriculture may not be viewed as an economic or dietary priority, but rather as a secondary or complementary activity [21]. Although IoT-based systems reduce the need for constant supervision of the crops and enable remote operation, few gardeners have the time or space to remotely monitor and control their greenhouse during their work day [4]. These factors are often overlooked in the design of IoT-based systems for urban gardening, reported in the literature.

*Technology adoption in a context of limited access to ICT*. Key challenges in designing technology for gardeners in Latin American cities include limited access to advanced technologies and a lack of familiarity with complex information systems [17]. The research suggests that for IoT-based monitoring and control systems to be adopted, they must be user-friendly and accessible, even to users with minimal prior technological experience [17].

*Ad hoc designs for Latin American urban gardeners*. Solutions for the Latin American context should be tailored to the specific realities of urban gardeners, rather than adapting systems designed for countries with different social, economic, or environmental characteristics [4]. In many cases, urban gardeners in developed countries may have greater access to technological and financial resources and a higher rate of ICT adoption. In contrast, many urban gardeners in Latin America lack the resources to invest in advanced systems or have limited access to technological infrastructure [3]. Cultivation areas often implement partial automation due to the cost of this technology; therefore, support systems should be designed to scale modularly.

*Differences from other international urban gardening contexts*. Cultural and social differences among urban gardeners worldwide influence both the feasibility of the activity and how it is conducted. In Western countries, gardening is viewed as a leisure activity that promotes wellbeing [22,23]; however, in developing countries, it primarily represents a source of food for households [21]. In countries like China, urban rooftop farming is a planned, large-scale practice supported by public policies and advanced infrastructure [24]. Similarly, in cities in Europe or the United States, urban gardeners often have greater financial and technological resources, allowing them to adopt more sophisticated systems for monitoring and controlling their crops [25]. In contrast, urban farming in Latin America is typically small-scale and family-based, focusing on subsistence or improving local food security. In this context, technological solutions must be tailored to the space and resource limitations of these gardeners [3].

### 2.2. Hydroponic Cultivation in Greenhouses

Hydroponic cultivation requires a nutrient solution (i.e., water mixed with nutrients) and a medium that supports the plant while allowing the roots to oxygenate and absorb nutrients properly. Coconut fiber and rice fiber are the most used materials for this purpose due to their ability to retain water, maintain pH levels, and provide oxygen to the roots of the vegetables [13]. This eliminates difficulties related to soil quality, helps prevent or reduce diseases and fungi, and decreases the use of insecticides and pesticides [26]. Due to the accurate control of the environment required in the productive sector, this cultivation style is performed in greenhouses. It also allows for the extension of the production periods [27] and the use of small spaces, such as rooftops or balconies. 

If the greenhouse is equipped (e.g., controlled using an IoT system), the environmental conditions for growing vegetables can be optimized [28]. This significantly simplifies and enhances the flexibility of gardening activities [4]. However, gardeners must still manage specific aspects of certain vegetables. For instance, high temperatures and humidity inside the greenhouse create an environment conducive to the development of diseases; in the case of tomato cultivation, this can include mildew [20]. Detecting these diseases using sensors or image processing can be challenging and may require complex and expensive technology [18]. Therefore, IoT systems designed to support cultivation in greenhouses should complement gardeners rather than replace them. 

The literature reports the cultivation experiences of various vegetables, including lettuce, chard, spinach, tomato, cucumber, and onion [20]. Moreover, recent research demonstrate the feasibility of growing small trees [29]. This opens up several options for urban gardeners to grow fruits and vegetables at a family scale.

## 3. Related Work

The literature related to hydroponic cultivation using greenhouses and IoT-based solutions is large and diverse, but few works specify the cultivation context for which a system was designed or recommended. This context includes variables such as the cultivation scale, the gardeners’ culture, their knowledge to perform the activity and use technology, the system’s initial costs, and the activity’s purpose (e.g., commercial, leisure, social, or food for households) [21,22,23,30,31]. Depending on the cultivation context, a system may be suitable for some situations but unsuitable for others. 

While the literature acknowledges the influence of the cultivation context on the suitability and adoptability of support systems, most reports on hydroponic cultivation leave the responsibility for the system design and technology selection to the designers’ criteria. In that sense, the literature reviews (e.g., [29,32,33,34]) typically focus on analyzing alternatives to implement the four layers of an IoT system [35]: sensing, data transport, data processing, and application for end-users. 

The first three layers of this architecture represent the monitoring and control system (MCS), and the application layer corresponds to the software system utilized by the end-users (in this case, the urban gardeners). The MCS is in charge of monitoring and controlling the variables that represent the cultivation conditions [36,37], and the software application (usually a mobile app) allows the gardener to interact with the MCS, typically to receive information and eventually send commands to specific components of the system (actuators) as needed. Next, we analyze the related work on these components, particularly considering their adoptability by different populations.

### 3.1. Monitoring and Control Systems

The literature presents a wide variety of MCSs designed to support hydroponic cultivation in greenhouses. Most of this research focuses on improving the structure or the operation of these systems; for instance, reducing water and electricity consumption [4,38], or optimizing irrigation times [39,40]. 

Some of these MCSs incorporate complex mechanisms, often based on artificial intelligence, e.g., determining the best time for harvesting [41,42], assessing and forecasting the quality of nutrients [43,44], or providing recommendations to optimize the cultivation parameters [45,46,47]. There is also a significant number of articles that report systems designed to cultivate specific types of vegetables [7,8], fruits [46,48], and microgreens [43]. 

On the one hand, the previous works show that several systems can adjust their behavior (through configurations) to maintain particular cultivation conditions, and thus to address the production of several vegetables and use different cultivation infrastructures. On the other hand, as with any other ICT system involving human end-users, MCS are designed to account for the particularities of a specific population (as potential adopters), for instance, urban farmers (for commercial purposes), urban gardeners (with non-commercial purposes), or community growers who also have particular socio-economic conditions to be addressed. 

Therefore, the feasibility of adopting a certain MCS by a particular population is directly influenced by their ability to acquire and operate it, while also considering operation costs [12,13]. In that sense, the MCS conceived for use in developing countries (i.e., that involve low acquisition and operation costs), like those reported in [49,50,51], could be used in Latin American urban gardening scenarios. Moreover, in several Western and Asian countries, local governments provide financial support to gardeners and farmers to promote urban agriculture [52,53], which positively influences the adoption of these technologies.

Even if the gardener is able to acquire the MCS, another major barrier to effectively adopting a given support system remains. This barrier is the adoption of the software application that complements the MCS. Typically, the MCS and mobile application are a combo that should be adopted by the gardener. Therefore, these components, both individually and collectively, should be suitable for end-users.

### 3.2. Applications for Gardeners 

Software applications to monitor hydroponics (usually a mobile application) receive the messages from the MCS and inform the end-user about events occurring in the greenhouse. In designing the application, a significant opportunity to consider the economic, cultural, and social aspects of the gardeners presents itself. Such a design strongly influences the usability and usefulness perceived by the end-users, and therefore, the adoption of these systems [14,15,16].

Almost all MCSs rely on a mobile application for the gardener, but few have been reported in the literature about how to design them to improve their usability and usefulness for a specific population. The lack of consideration of these design aspects may help explain the significant differences in the adoption of hydroponic urban gardening technology observed across various regions and cultures [10,11]. The acquisition and operation costs of the system, its capability for incremental extension, and the language used by the application to interact with the gardener are the first inclusion/exclusion criteria used by Latin American gardeners to identify potential technologies to adopt to support their activities.

Considering the socio-cultural aspect as a factor influencing technology adoption [12,14], the mobile application utilized by the gardeners influences this adoption as much as the MCS, but from a different perspective. Particularly, the behavior of a software system (even the MCS) can be conceived to be context-aware, allowing for self-adaptations of its services based on changes in the gardeners’ context. The literature shows some proposals that consider the context of the cultivation area, e.g., [54,55], but none of them take into account the gardeners’ context; for instance, their workday, free time, routines, or simply their preferences. 

In urban gardening performed at a family scale, considering the gardeners’ context makes a difference in the suitability and adoptability of these systems. This opens an opportunity to explore the suitability of these systems considering socio-cultural scenarios, and thus designing IoT-based systems more appropriate for the end-users. Policy makers, crop-science researchers, and designers of IoT systems can take advantage of this.

## 4. The Conceptual Design of the System

**Figure 1:**
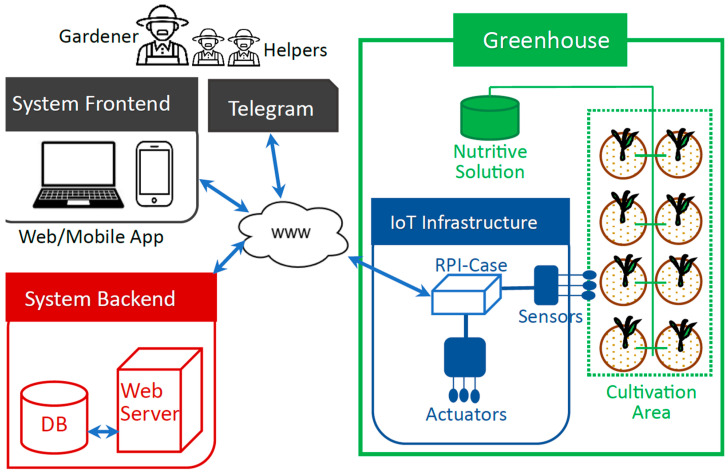
The operational environment of the IoT-based hydroponic cultivation system.

The system involves three major components (Figure 1): the greenhouse, the IoT infrastructure, and the software system that includes a frontend and a backend. Each component plays a specific role; for instance, the greenhouse contains the cultivation area, the crops, and a tank with the nutritive solution necessary for hydroponic cultivation. Plants in the cultivation area are grown in plastic buckets filled with a coconut-fiber substrate and a nutrient-rich water solution. This area is monitored using the IoT infrastructure (shown in blue in Figure 1). This infrastructure periodically sends sensor data to the server. Additionally, the infrastructure executes commands received from the system’s frontend and backend. 

The backend manages data and autonomously makes decisions to maintain optimal cultivation conditions (e.g., turning an actuator on or off), while informing the gardener of situations that may require their intervention; these notifications are sent through Telegram messages. 

The system supports two end-user profiles, gardener and helper, because the cultivation process (even at a small scale) tends to be a collaborative activity in Latin American culture. The gardener (with the possibility of more than one per greenhouse) is able to use the frontend application to monitor and control the cultivation variables in the greenhouse. These users also receive Telegram messages from the system’s backend control component. 

The gardener can have helpers to support activities in a particular greenhouse. Helpers can only receive and respond Telegram messages from the backend or the gardener. Helpers are usually members of the gardener’s family, who are asked to carry out tasks on-site in the greenhouse as needed. For example, on a windy day, the backend control component might request a helper to check the wind intensity on the rooftop where the greenhouse is located. This request might be triggered when the gardener is unavailable, and ventilation is required in the cultivation area due to the high greenhouse temperature. However, the backend temporarily refrains from opening the window until confirmation is received from the gardener or helper, as high wind intensity could damage the greenhouse. This example demonstrates how helpers’ involvement can be crucial, especially in greenhouses that are not fully automated, as is often the case in Latin American urban settings. 

Concerning the system’s frontend (i.e., the web/mobile app.), it provides a user interface for the gardeners where they can request the historical or current values of variables recorded on the server. These variables include those monitored and controlled within the greenhouse (e.g., temperature, electrical conductivity, and pH), and other context-relevant factors, such as actuator status and the weather forecast for the next two hours. Moreover, this application allows the gardeners to send on-demand commands to sensors and actuators and set acceptable ranges for controlled variables. These commands are always routed through the system backend, which is responsible for recording the status of cultivation variables and IoT infrastructure components. 

In general terms, the architecture of the operational environment does not differ significantly from others reported in the literature for this or similar purposes, as seen in [8,35,49]. A similar situation applies to the monitoring and control system (shown in blue in Figure 1). However, what sets this system apart from previous ones is primarily its capability to be configured, adjusted, and extended to meet the specific needs of particular gardeners, e.g., urban gardeners in Latin America. This capability is a result of using the blackboard design pattern [56] for modeling the system backend. This allows for the customization of the behavior of this component almost in real-time, based on the rules specified as data in the configuration files that represent both the cultivation and the gardener’s contexts. 

This self-adaptation capability, which is not present in other proposals, could also be valuable for crop-science researchers, and IoT systems designers, because it enables the rapid prototyping of system behavior by modifying rules in a configuration file. This strategy for the behavioral self-adaptation of an IoT-based system can be reused by other systems. 

Another novelty introduced in this work, which is part of the gardener context, is the inclusion of helpers as formal roles to complement the gardener and the IoT-based system. This role comes from the Latin American culture, and it is highly relevant for supporting cultivation activities in partially automated greenhouses, such as the ones addressed in this work. 

**Figure 2:**
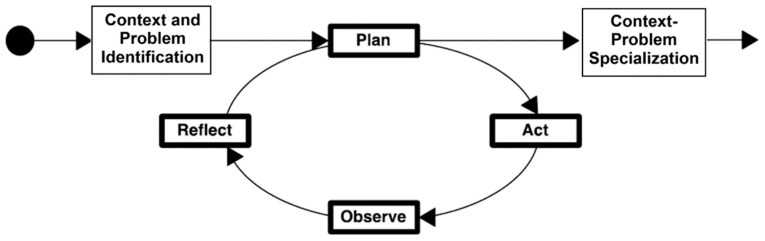
The structure of the action-research approach.

The system and its conceptual design were developed and evolved using an action-research (AR) approach [57]. This approach allows for performing one or more active in-the-wild interventions in the studied scenario (Figure 2), and it is particularly valued when the goal is to observe the effects of introducing a new technology or method into the study population in depth [58]. Based on the empirical results of these interventions, technology designers can refine and evolve their artifacts even in application scenarios that involve high uncertainty [59], such as urban gardening in the study domain. In the next section, we describe the implementation of the major components of this system, which is the result of the third AR cycle.

## 5. System Implementation

**Figure 3:**
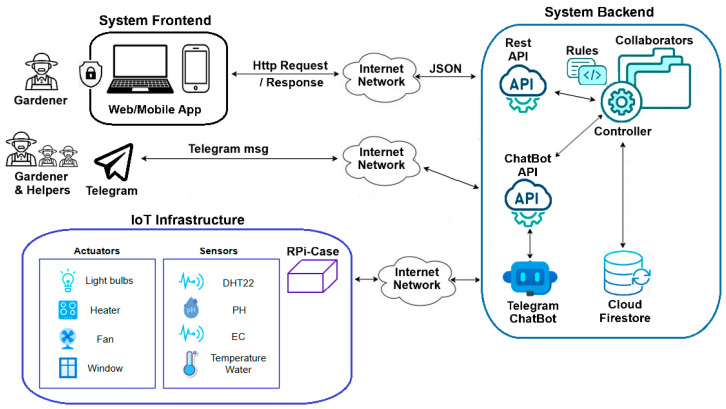
The multi-tier architecture of the software solution.

The system implementation follows a multi-tier architecture; specifically, a three-tier + service structure as indicated in [60]; this consists of presentation, service, business, and data-access layers. Figure 3 provides a detailed illustration of this architecture. 

The *presentation* layer is implemented by the system frontend. The *service* layer is implemented through two APIs within the system backend: the Rest API for the controller and the Telegram ChatBot API. The system controller constitutes the *business layer*. It utilizes services provided by the service layer (i.e., API endpoints) to interact with the frontend and IoT infrastructure. These components, along with Telegram, are treated as remote services. Finally, the *data-access* layer is implemented on Cloud Firestore. This modular, scalable architecture enhances the code organization, readability, and maintainability of the application. 

Typically, end-users (i.e., the gardeners) log in to the system through the frontend (presentation layer), allowing them to request data from the system backend (business layer) or send commands to the IoT infrastructure (remote service) via APIs (service layer). This setup enhances communication efficiency and security among components. Responses to end-user data requests are returned in JSON format. Data input from external sources, i.e., the frontend and IoT infrastructure, is consistently managed via the Rest API (service layer), with data persistence handled by Cloud Firestore (data-access layer). The following sections detail each of these layers.

### 5.1. Presentation Layer: System Frontend

Only authenticated users have access to system services. User authentication relies on JSON Web Token (JWT), an open standard (RFC 7519) that represents structured and digitally signed information in JSON format. These JWTs are securely transmitted between the sender (e.g., backend) and the receiver (e.g., frontend), enabling both authentication and trusted information transmission. 

Through the frontend, the gardeners can monitor greenhouse variables, or trigger commands to operate system actuators. These actions can be performed via the system frontend or through Telegram.

**Figure 4:**
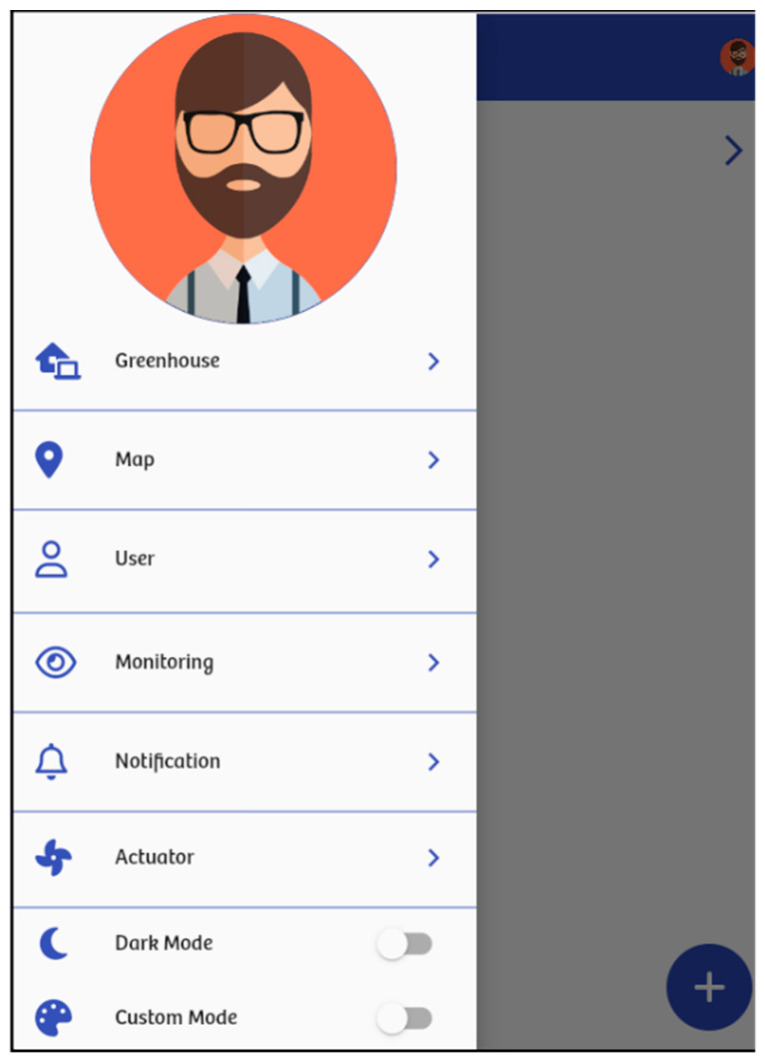
The website menu for monitoring. The user interface of the web/mobile application were originally in Spanish; they were translated to English for comprehensibility.

Developed in Flutter, the system frontend can be deployed as a web application or a native app across multiple operating systems and devices. This application’s main menu offers seven options (Figure 4): greenhouse, map, user, monitoring, notification, actuator, dark mode, and custom mode. 

The “greenhouse” option displays a list of greenhouses registered in the system in which the logged-in user is involved. Moreover, registered users can create new greenhouses or select an existing one to monitor.

The “map” option displays the greenhouse location via Google Maps, and the “user” option enables the greenhouse owner (i.e., the creator) to manage gardeners with access and designate helpers to receive Telegram messages. 

**Figure 5:**
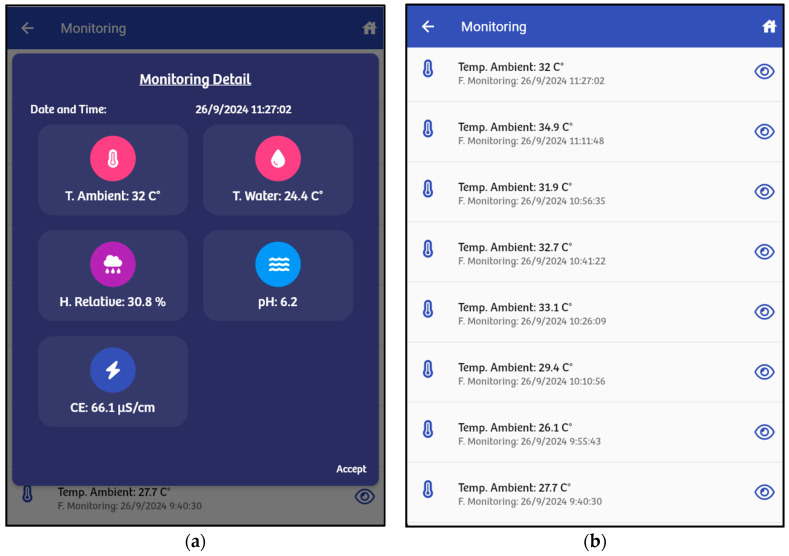
(**a**) The current values of the monitored variables; (**b**) detailed temperature information (monitored variable).

After selecting a greenhouse, the “monitoring” option lets users retrieve monitoring information for a specific time period. Here, users can view the current values of the variables (Figure 5a) or the details of specific variables such as temperature (Figure 5b). 

The “actuator” option enables the management of actuators, including the heater, light bulb, fan, and window. The heater and bulb activate when the greenhouse temperature drops below a set limit, e.g., less than 15 °C, and deactivate once it exceeds 15 °C. The fan and window activate when the greenhouse temperature rises above 28 °C. Similarly, when the temperature falls below 28 °C, the fan turns off and the window closes. These ranges are set by the gardener based on the crop’s requirements.

The “notification” option displays a list of warnings or alerts generated by the system. Notifications are triggered when a variable’s current value falls outside of the acceptable range. Table 1 outlines the acceptable ranges for tomato cultivation. 

**Figure 6:**
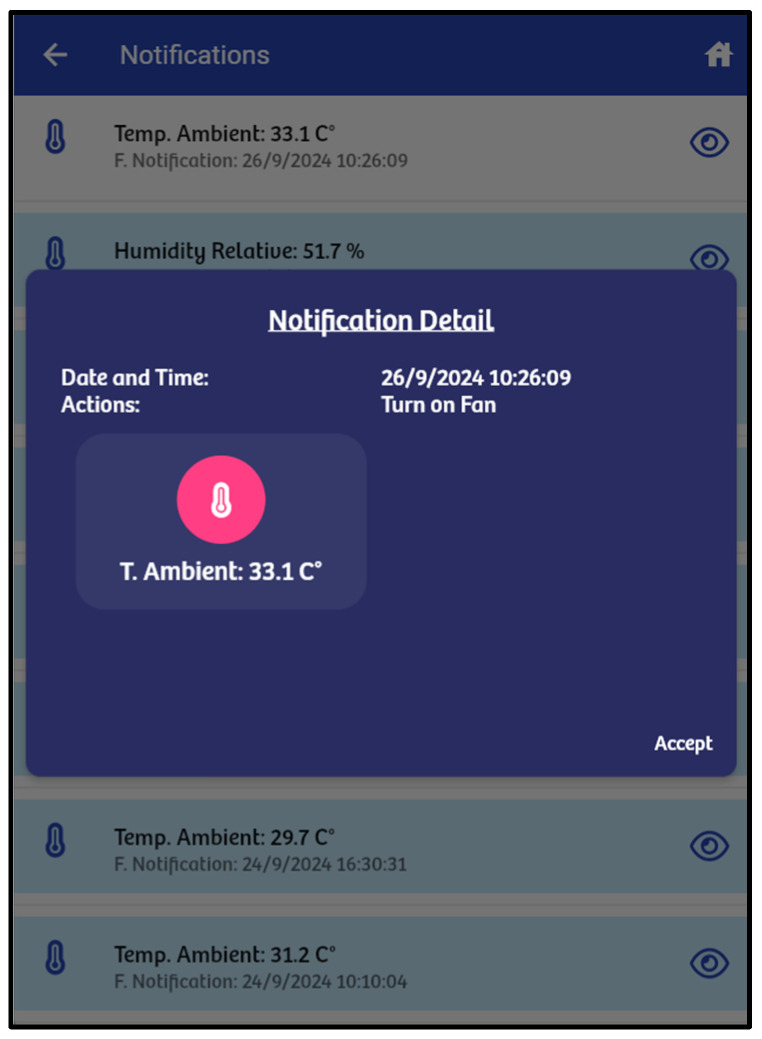
Temperature warning.

**Figure 7:**
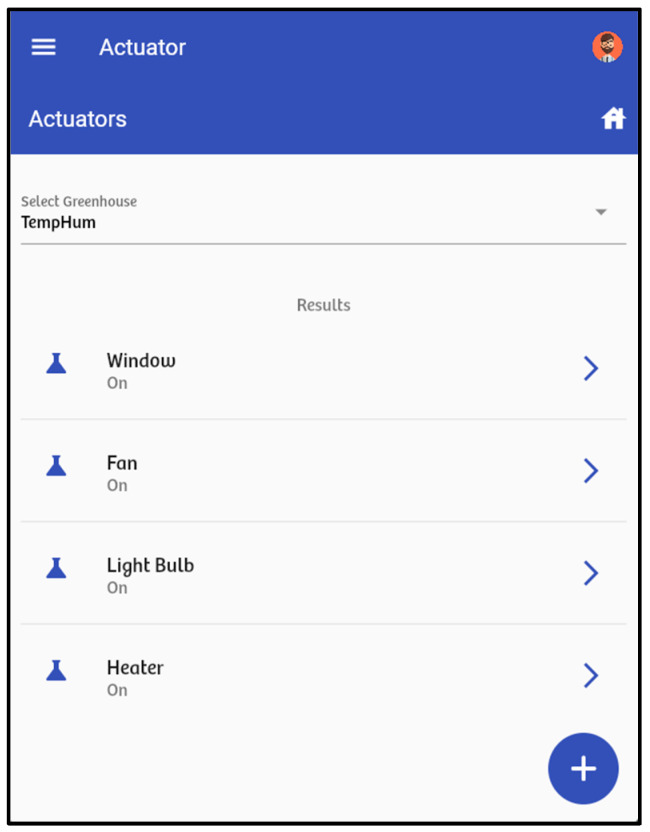
List of actuators deployed in the IoT infrastructure.

Figure 6 shows an example of a notification sent to the frontend when a variable, in this case temperature, is outside the acceptable range. Figure 7 displays the list of available actuators in the IoT infrastructure, their status, and any associated notifications.

### 5.2. Service Layer: Rest and ChatBot API

The service names for both APIs were standardized to simplify their use during the system’s development and evolution. The Rest API includes endpoints that control the IoT infrastructure (Table 2) and those required to manage the greenhouses and users (Table 3). 

Using Telegram messages enables the user to log in from multiple devices and interact with the system in various ways, e.g., to request specific variable values, remotely control actuators, and take on-demand photos or videos to monitor vegetable growth. Table 3 shows a sample of endpoints implemented in the Rest API, which complements the previous API.

### 5.3. Business Layer: System Backend

**Figure 8:**
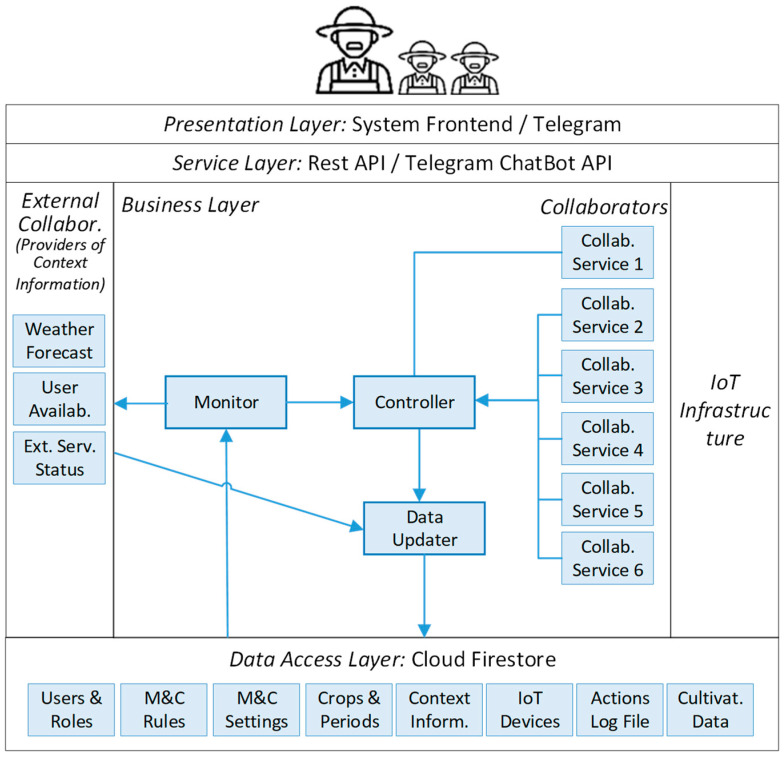
The architecture of the system backend.

The system backend implements a blackboard pattern [56] using data space provided by the data-access layer. This pattern includes three components: the blackboard, the knowledge sources, and the control. The *blackboard* represents the global memory that contains the data entities managed in the solution space. In this case, this role is played by the data repository implemented in the Cloud Firestore database, which is part of the data-access layer of the system architecture (Figure 8). 

The *knowledge sources,* also known as collaborators, are components specialized in performing activities to generate new information that contributes to solving a problem or answering a question. For instance, one of these components could oversee forecasting the evolution of the wind intensity and direction for the next two hours in the area where the greenhouse is located. The information generated by these components increases the data space available to make decisions, usually about how to keep the cultivation variables under control.

The knowledge-source components perform their activities only when the controller asks them. The *controller* is an autonomous component, and its main goal is to make decisions when required, using the information available on the blackboard. To do that, the controller uses more than just the knowledge sources as helpers; for instance, the external collaborators and the monitor component. The external collaborators are providers of contextual information that can be used to complement the data stored on the blackboard. For instance, in the case of an electrical power outage, the electricity provider can inform the user (through a software service) of the time at which the electrical power will be reestablished.

The controller also uses the monitor as a helper to identify situations in which it must make decisions. The monitor is also an autonomous component, which is continuously analyzing the information in the blackboard, considering the monitoring details and controlling the rules also available in that component (the rules are specified in an XML file). When the monitor detects or envisions a situation, it notifies the controller to act. A copy of the rules file is stored in the local controller of the IoT infrastructure (RPI-Case in Figure 3); it allows the RPI-Case to maintain control of the infrastructure during periods in which the Internet service is temporarily not available. 

To maintain the separation of concerns among the components implemented in the business layer, the updates of the information of the blackboard are performed through the data updater. In case of conflicts or potentially incorrect information, this component decides whether to update the blackboard.

### 5.4. Data-Access Layer: Data Repository

As previously mentioned, the blackboard was implemented using a non-SQL database (Cloud Firestore). This database represents the data entities for a greenhouse, shown in Figure 8. Table 4 briefly explains each entity. 

### 5.5. External Service: Monitoring and Control of the IoT Infrastructure 

**Figure 9:**
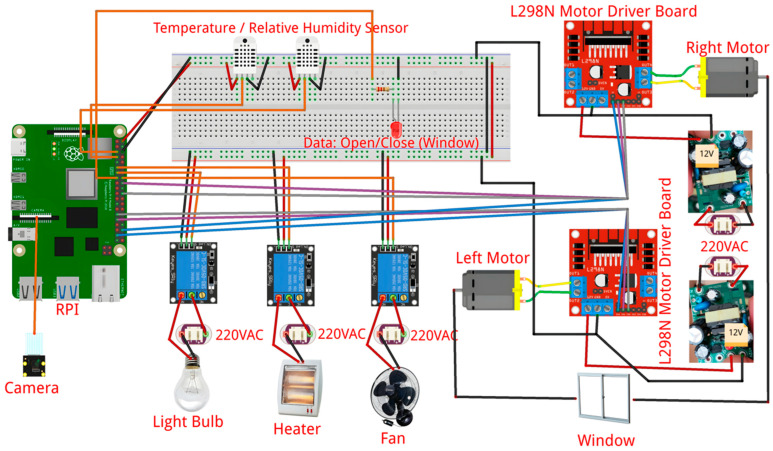
IoT infrastructure of the MCS (RPI-case).

The IoT infrastructure in the greenhouse is managed by a control box (labeled as RPI-Case in Figure 3), containing a Raspberry Pi and software to control the sensors and actuators. This box is connected to environmental sensors for temperature and relative humidity, as well as sensors that measure the electrical conductivity, the pH of the nutrient solution, and the temperature of the irrigation water. The control box can manage five types of actuators: video camera, light bulbs, heaters, fans, and windows. Figure 9 shows the architecture of the monitoring and control system (MCS), highlighting the electronic devices used in this prototype and the connections required between components for this infrastructure to operate. 

**Figure 10:**
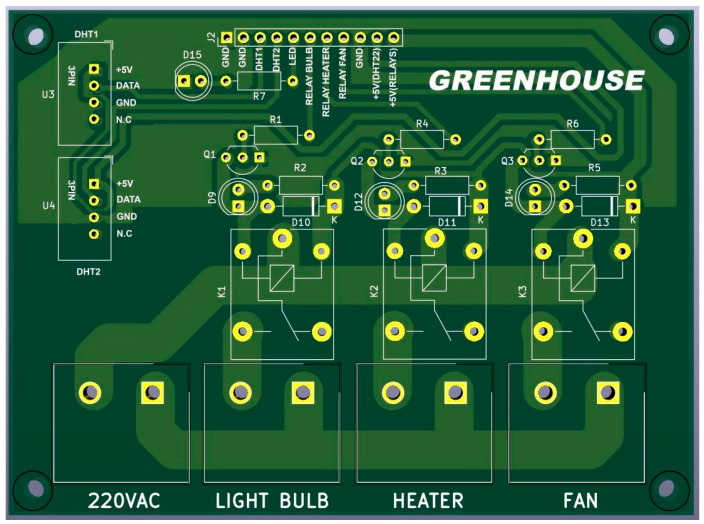
Printed circuit board of the RPI.

The components of this infrastructure are managed by software embedded in the Raspberry Pi, which uses the same configuration file as the system backend. The greenhouse components are modular; therefore, the RPi operates based on the sensors and actuators that are connected and registered as active. Figure 10 shows the PCB of the RPi.

At startup, the software verifies that the sensor data falls within acceptable crop parameter ranges. If any value is out of range, the backend sends a notification to the gardener and initiates the corrective actions; these actions are managed by the MCS when Internet service is available. To execute these actions, the system activates the appropriate actuator (heater or fan) via a relay, or opens/closes the window according to a preset command sequence in the configuration file. 

The IoT infrastructure also considers a video camera that is connected to the Raspberry Pi. The camera interface functionality can be verified using raspi-config. The L298N module is a dual H-bridge that controls the rotation and speed of a stepper motor to open/close the window. 

The RPi retrieves data from the sensors and sends them to the server, which stores them in a database, making them accessible for querying and decision-making. Both the gardener and the system utilize these data. Next, we describe the system’s main components and outline the acquisition and operation costs. 

#### 5.5.1. Sensors and Controlling Devices

Table 5 lists the sensors and control devices used in the IoT infrastructure. The DHT22-1 and DHT22-2 sensors connect to the GPIO (General-Purpose Input/Output) port, using pins 4 and 3, respectively. This sensor type has a GND (Ground), a VCC (Voltage Common Collector), and data pins, which output serial data signals for temperature and relative humidity.

The EC, pH, and water temperature sensors (from Atlas Scientific EZO Circuits) connect to the tentacle T3 module for Raspberry Pi. The monitored variable values are retrieved using the addresses assigned by the AtlasI2C library. The default addresses are 99 (PH), 100 (EC), and 102 (TempWater).

#### 5.5.2. Actuators

The IoT infrastructure includes several actuators. Specifically, the light bulbs, heater and fan are connected to the 220 V power supply and to a relay that has three inputs and three outputs. The required inputs for these actuators are GND, VCC, and the relay control. The outputs used are COM (common pin) and NO (normally open). The light bulbs are connected to GPIO-27 of the relay control, the heater to GPIO-17, and the fans to GPIO-23. This setup enables turning these actuators on and off.

The window, which facilitates the greenhouse ventilation, is connected to a L298N motor driver module, enabling the control of two 12 V motors. The L298N motor is connected to GPIO-26, GPIO-6, and GPIO-5, and the other motors to GPIO-16, GPIO-24, and GPIO-25. These motors rotate clockwise or counterclockwise to open or close the window, respectively. Additionally, the system includes a LED and a resistor connected to the GPIO-22 to indicate whether the window is open or closed.

#### 5.5.3. Acquisition and Operation Cost of the System

The IoT infrastructure components cost approximately USD 220 if purchased in Peru, with itemized prices as follows: pH probe and circuit (USD 9.21), temperature sensor DHT22 (USD 4.5 × 2), CE probe and circuit (USD 66.32), Module L298N (USD 2.6 × 2), Raspberry Pi 4 (USD 42.37), heater (USD 20.53), fan (USD 23.68), light bulb (1.32 × 2), relay (2.63 × 3), stepper motor (USD 9.21 × 2), water temp sensor (USD 4), protoboard, cables and others (USD 4), and the wood case (USD 3.95). While the software for monitoring and control is free and available online (https://temphum.com.pe/ (accessed on 27 November 2024)), additional costs arose from integrating these components into the RPI-Case. The mobile/web application used by the gardeners is also freely available at the Google Play Store (the TempHum app).

For continuous operation, monthly energy costs depend on several factors, mainly the temperature difference between the greenhouse’s interior and exterior, which affects the energy needed to maintain optimal conditions for plant growth. Temperature control, especially during winter when the heater is frequently in use, is the most energy-intensive aspect of the system. 

During this test period, peak energy consumption was 93 kW in Sept 2023 (USD 16 in Peru), while the lowest consumption was 28 kW in November 2023 (USD 4.8). The Internet connection costs were considered as zero, due to almost any home already counting on such a service in urban zones; this was a positive consequence of the pandemic. The water consumption is around USD 3 per month.

These costs are non-negligible, and they appear affordable for a substantial portion of the urban population. According to prior research [3,17,18], the main challenges for adopting this technology lie in its ability to accommodate the socio-cultural aspects of gardeners’ routines. 

### 5.6. Software Technology and Development Approach

The system reported in this article has been used and improved since 2020, when it was first used to cultivate lettuce [49]. Following an action-research approach [58], two additional improvement cycles were conducted to enhance the system’s suitability for supporting urban gardeners in Abancay city, Peru. Various gardeners participated in these previous experiences, during which the system was used to cultivate strawberries, goldenberries, parsley, and two varieties of tomatoes. The current version of the system (4.0) is implemented using the languages, libraries, and tools listed in Table 6.

### 5.7. System Preliminary Evaluation

Before outlining the experience reported in the next section, we conducted a preliminary evaluation of the new version of the system, to reduce uncertainties about the system’s suitability for potential users. A black-box evaluation process was carried out to monitor and control a real greenhouse on-demand. The evaluators utilized the mobile application to monitor variables in the cultivation area, trigger commands to control the actuators, and perform setup activities (e.g., to create a greenhouse and assign gardeners and helpers). Then, the participants completed the CSUQ scale [61] to assess the perceived usability and usefulness of the system, as well as the quality of information it provides. 

#### 5.7.1. The Participants

The recruitment process began by sending an open invitation via email to members of the community of the Micaela Bastidas de Apurímac National University, located in Abancay, Peru. This invitation included students, as well as the academic and administrative staff of the institution. The invitation specified that participants should have at least one year of experience in urban farming or gardening to take part in the evaluation process. 

The evaluation process was performed in two rounds. In the first round, thirty-five individuals responded to the email, expressing interest and confirming that they met the inclusion criteria. After scheduling the date and time for the evaluation experience, twenty individuals ultimately participated in this exercise: twelve men and eight women aged between 32 and 57 years. In the second round, twenty people responded the email, and after confirming the meeting, twelve individuals participated: five men and seven women aged between 28 and 45 years. This totaled 32 participants: 17 men and 15 women.

All participants had at least two years of experience in urban vegetable cultivation in greenhouses, and six of them had cultivated vegetables using hydroponics. 

According to the recommendations reported in [62], twenty participants is the minimum number required to perform an evaluation of a software system based on “field observation, i.e., the users utilizing the application. Nonetheless, the purpose of this evaluation was to determine whether this new version of the system was ready to be used in a new cultivation experience. 

#### 5.7.2. Evaluation Protocol

First, the reviewers were introduced to the system for 15 min, after which they were required to complete five tasks on an already implemented greenhouse: (1) to create an extra user for such a greenhouse, (2) to identify the status of the monitored variables (temperature, humidity, EC, and pH), (3) to monitor the temperature and humidity over a specific time period, (4) to check the current status of the actuators, and (5) to check the notifications sent by the system regarding two variables. 

Most participants continued to explore the capabilities of the system for a brief period (5–10 min) after completing the activities. Additionally, they voluntarily shared their feelings and suggestions immediately after performing the test. They then completed the CSUQ scale survey using a web form. Responses were anonymous.

#### 5.7.3. The Assessment Instrument

The instrument consists of 19 items that users should rate on a 7-point Likert scale (1: strongly disagree, 7: strongly agree). Items 1–8 rate “system usefulness”, items 9–15 evaluate the “quality of the information” provided by the system, and items 16–18 establish the “system usability”. Item 19 is open for qualitative comments. Table 7 lists the items of the CSUQ scale.

#### 5.7.4. Evaluation Results

All participants successfully completed the activities within 7–15 min. The average scores calculated from the CSUQ scale results were the following: 6.05 for the system usefulness dimension, 6.07 for information quality, and 6.20 for the system usability. Several participants gave comments like these: “the system is suitable enough to be used in real experiences”, “I would like to use it in my greenhouse” and “the app was intuitive and easy to use for me”. Given these preliminary results, and the maturity level shown by the system in the previous action-research cycles, we decided to conduct the experience described in the next section.

## 6. Experience Report

**Figure 11:**
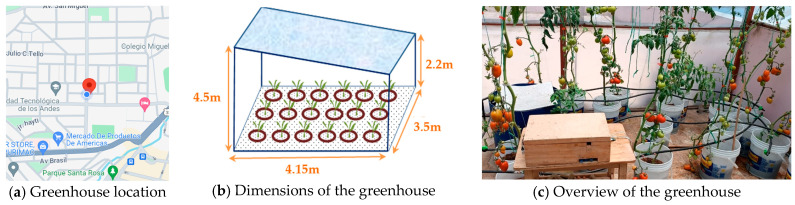
The greenhouse used in the cultivation experience.

This urban farming experience took place from September 2023 to March 2024 in an urban greenhouse located on the rooftop of a house in Abancay, Peru. The greenhouse was located at 508 Panama Avenue (Lat: −13.6323046804, Long: −72.885837664), in the downtown of the city (Figure 11a). 

Figure 11b shows the design of the greenhouse; it has an area of 14.53 m^2^ and a volume of 48.68 m^3^. Eighteen plastic buckets were arranged in the greenhouse to cultivate tomatoes using hydroponics (Figure 11c); eleven were planted with Roma tomatoes (one plant per bucket) and the other seven were planted with Globe tomatoes. Coconut-fiber substrate was used to support the plants throughout this cultivation process.

### 6.1. Main Goals of This Experience 

It is important to note that maximizing tomato production or optimizing cultivation variables, such as production time or the use of water for irrigation, was not a goal in this experience. Instead, we observed the system’s ability to adjust according to the gardeners’ needs; particularly, considering their sociocultural aspects that could promote or jeopardize the adoption of this technology. We recognize the diverse realities of urban gardeners, even within a single city. Therefore, we argue that such diversity should be considered in the design of the support systems to make that technology adoptable by urban gardeners. 

On the one hand, the socio-cultural aspects of the users usually provide valuable design insights to conceive these solutions. On the other hand, embedding self-adaptive capabilities into the systems and considering the user needs or preferences (i.e., the gardeners’ context) will enhance their adoption and provide personalized support to the end-users. The following questions were used to guide the exploration performed in this cultivation experience:

1. *Will a regular gardener be able to complete the whole cultivation process using the system?* This question does not aim to identify failures in the system, but to address differences between what the gardener needs to do, and what they can perform.
2. *Are there socio-cultural (or particular) aspects of the gardeners that should be considered in the behavior of the system to make it adoptable or more effective?* This question does not intend to identify all these aspects, but rather highlight some of them and emphasize the need to study them to transform how the support systems are conceived and designed.
3. *Is the proposed system able to self-adapt its behavior in a simple way to consider the temporary or permanent needs of the gardeners?* Recognizing that a wide range of adaptations could be required, this experience aims to determine what can be achieved by the current system in this sense.

### 6.2. Participants 

The cultivation activities were conducted by an individual knowledgeable in urban gardening, but not specifically on hydroponics. Therefore, this gardener received brief training on this type of cultivation and on setting up the IoT system. The entire instruction process took a couple of hours. 

The cultivation process was monitored by a member of the research team who acted as an observer. This person had physical access to the greenhouse and to the system, with a user profile of “gardener”. 

In the middle of the cultivation period, in particular at the flowering stage, three people unexpectedly became helpers: two teenagers and an older adult. They expressed their interest in voluntarily participating in this experience, motivated mainly by curiosity. These people had physical access to the greenhouse and accessed the system with a user profile of “helper”.

### 6.3. Deployment of the System 

The greenhouse used in this experience required minimum adjustments to take advantage of the MCS capabilities; this involved opening and closing the window using the system, in particular. After performing these adjustments, the gardener initiated the germination process. During that period, which lasted approximately one week, the gardener manually monitored the temperature and humidity in the greenhouse, and the temperature, humidity, electrical conductivity, and pH in each bucket, which contained the coconut-fiber substrate and a nutrient-rich water solution. This activity was performed twice a day, usually in the morning and afternoon after the gardener’s workday. The gardener used the multi-parameter Hanna device to measure the variables; then, they recorded the values in a paper notebook. If it was necessary to turn on/off an actuator, that action would be performed manually.

After the germination stage, each plant was transferred to a larger bucket to continue the cultivation process, and the gardener installed the MCS (i.e., RPI-case) and plugged in the sensors and actuators. These activities were monitored by the observer, who offered support to the gardener; however, the gardener did not need external assistance to complete them successfully.

Once the MCS was installed in the greenhouse, the gardener continued visiting and performing visual inspections of the tomatoes, but these activities were now perceived differently. In this sense, the gardener indicated, “*during the first week, the monitoring process was a bit stressful and time-demanding due to my low experience in hydroponics and the need to perform manual activities. After installing the system everything was more comfortable for me, because I realized that the system would also take care of the plantation*”. 

### 6.4. The Tracking of the Cultivation Experience 

**Figure 12:**
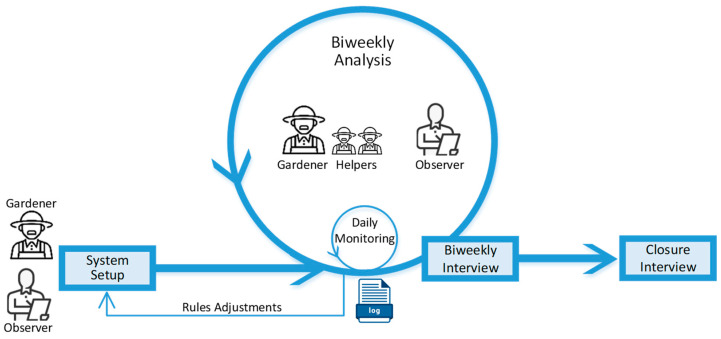
The monitoring and control of the cultivation experience.

The tracking of the cultivation activity followed the process shown in Figure 12. First, the gardener defined the values of the cultivation parameters (variables and ranges of acceptable values) and for their work context, e.g., the workday and regular periods without physical access to the greenhouse. This configuration process provided the initial information for the system to operate. 

Then, the observer used the mobile/web application every day to monitor the cultivation variables (indicated as “daily monitoring” in Figure 12), and occasionally recorded annotations in the log of the cultivation experience. The progression of the monitored variables and the dynamics of the actuators were reviewed weekly by the observer using the system.

Every two weeks, the gardener was asked about the growing process, how the system was functioning, and whether they had new requirements for it. The information resulting from these biweekly analysis activities was systematically recorded in the log. Every time the gardener had a new requirement, necessitating the adjustment of the system behavior, this was carried out by changing the system setup. Then, the observer used the log file of the system to verify that the solution (in particular, the MCS and the backend) effectively adjusted the system behavior.

The biweekly analysis activity also included a meeting with the helpers to understand their needs to perform the role, and to obtain their feelings about the experience. The resulting information was managed in the same way as the gardeners. These activities were repeated during the whole period. 

At the end of the process, each participant was interviewed to obtain a comprehensive opinion about the experience and perform a cross-check to validate the information gathered in the biweekly meetings. This information was also part of the cultivation log and was used to analyze the results of this experience. In the next section, we explain the cultivation stages that were part of this experience.

### 6.5. Tomato-Growing Process

**Figure 13:**
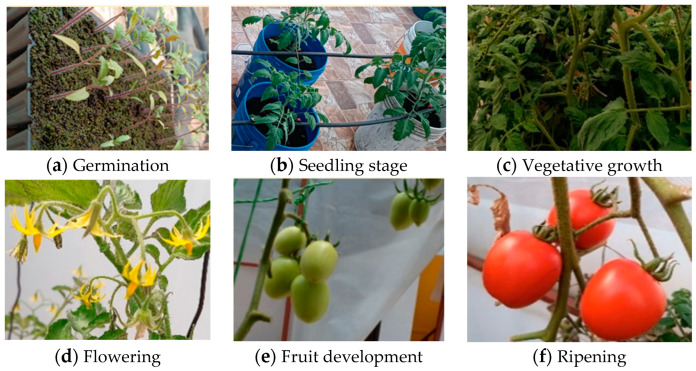
The status of the tomatoes in each growing stage.

The tomato cultivation process consisted of the following stages (Table 8): germination, seedling stage, vegetative growth, flowering, fruit development, and ripening. Table 9 presents the periods during which these stages occurred, along with images of the plants at each growth stage (Figure 13).

### 6.6. Tomato Production Results 

Table 10 presents the tomato yield achieved during this experience, which represents a tangible result. A total of 20.97 kg of Roma tomatoes and 20.85 kilos of Globe tomatoes were produced; there were 11 plants for Roma tomatoes, and 7 plants for Globe tomatoes. On average, 1.91 kg of Roma tomatoes and 2.98 kg of Globe tomatoes were obtained, which can be considered an average production amount for these varieties. 

The operational cost for the cultivation period (five months) was USD 39.7 for energy consumption and USD 15 for to water consumption. Therefore, the cost of producing one kilogram of tomatoes is around USD 1.3. This cost is a bit lower than the price of the commercial tomato (USD 1.5), and the difference is not large enough to impact the economy of a family. 

Similar to other reported experiences [23], the motivation to perform urban gardening at a family scale was usually not economic, but related to physical and mental health, and for social and educational reasons. Typically, the initial investment should be borne by the gardeners, hopefully with the support of government agencies. 

Concerning the first question stated in Section 6.1 (*will a regular gardener be able to complete the whole cultivation process using the system?*), which was part of the motivation to perform this experience, we can say that the gardener was able to (1) perform the whole cultivation process without stress, (2) produce an amount of tomatoes that is average for hydroponic greenhouse production, and (3) achieve a cost lower than that of the commercial tomato. Therefore, the results obtained are in line with what was expected at the beginning of this experience. 

According to what we observed, and also as indicated by the gardener, the main benefits of this experience were the intangible things, which are discussed in the next section. 

### 6.7. The Intangible Results of the Process

This cultivation process attracted the attention of two young family members, as vegetable growing is part of their middle-school curriculum. As a result, during the flowering process, they began to assist as helpers. The same occurred with an older neighbor of the gardener; therefore, they were added to the gardener’s profile as helpers in the cultivation. 

The system sequentially requested the assistance of these helpers on several occasions, e.g., when the electric power was temporarily unavailable. In these cases, a helper would check the temperature in the greenhouse and, if necessary, open the window. This collective participation transformed the perception of the cultivation process, shifting it from an individual initiative into a collaborative family project. 

After completing the cultivation process, we asked the gardener about his perception of participating in this experience. The most remarkable quotes were the following: “*… this experience makes me realize that hydroponic cultivation can be taken up as a hobby to perform at home, that also allows us to consume healthy products*” (…) “*I appreciate the reduction of time and stress involved in taking care of the tomatoes. However, I continue staying aware of them because the activity became a kind of therapy for me… it relaxes me*” (…) “*I also realized that this type of cultivation does not jeopardize my capability to perform other leisure activities*” (…) “*My project is now the family project. I enjoy listening to my kids talk about the crops during dinner and sharing this experience with me*”.

On the other hand, the three helpers indicated that they were happy to have participated in this experience. The older adult mentioned “Although my participation was required only a couple of times, it keeps me engaged. It was was much more interesting than watching TV”. 

When the helpers were added as the gardener’s support, we recorded their available and unavailable periods in the setting file, considering their daily routines; the same was done for the gardener. Then, we created a rule to use that information to determine if a person could be contacted by the system in a specific period (if required). The analysis of the log file indicates that the people were notified or asked for help only in periods when they were available, otherwise no information about it was recorded in the system. This demonstrates that the self-adaptation mechanism worked properly.

Moreover, creating and storing this new rule involved editing an XML file (the M&C Rules file) and took less than 5 min. It shows the system can change its behavior and self-adapt it to the users’ needs easily and quickly; this was the third question defined in Section 6.1 (i.e., *is the proposed system able to self-adapt its behavior in a simple way to consider the temporary or permanent needs of the gardeners?*). However, in this version of the system, this behavior adaptation still requires the participation of someone with technical knowledge to edit an XML file and write a rule using a particular syntax. Overcoming this limitation and making the rules specification addressable by regular users is part of the future work.

Concerning the second question (are there socio-cultural (or particular) aspects of the gardeners that should be considered in the behavior of the system to make it adoptable or more effective?), we found that considering the routine of the participants and their available time was appreciated by them. Moreover, in this case, the people that were invited to be helpers in the cultivation process were happy to accept the invitation and remained engaged in the activity until the cultivation process was concluded. This probably shows an important willingness of Latin American people to participate in community initiatives, including urban gardening. Technology designers and researchers in crop and social sciences could take advantage of this to conduct their own studies on gardening activities from different perspectives.

## 7. Analyzing the Features of Hydroponic IoT Systems to Support Urban Gardening

All systems discussed in the related work involve capabilities to sense variables in hydroponic agriculture scenarios, and most of them were designed to support cultivation at a small scale. Table 11 revisits the most relevant ones, and analyzes the features that influence the voluntary adoption of these IoT solutions by urban gardeners who perform this activity with non-commercial purposes. 

The monitoring and controls system (MCS) used by these solutions involve similar components; therefore, they are comparable in terms of the acquisition cost. In this sense, the systems reported in [40,47] are a bit different, and more expensive in terms of components, because they were designed to support cultivation at medium and large scales.

Concerning the variables that these systems can sense, and eventually control (column 3 in Table 11), they are aligned to what is usually required to cultivate particular vegetables at a small scale in greenhouses. The aspects that really differentiate these IoT solutions among them are (1) the software services provided to support the autonomous behavior of the system and the on-demand activities of the gardeners, and (2) the data these solutions record and use for improving the cultivation experience.

Columns 4 to 7 indicate the capability of the IoT infrastructure of each system to collect, store, process, and control cultivation variables. Many of them allow for autonomous sensing, others provide autonomous sensing and controlling, but only the proposed system provides these services in an autonomous and on-demand way. This capability allows the gardener to deliver orders to the actuators or ask for the current value of a certain variable.

Concerning the IoT system architecture and the software architecture (columns 5 and 6, respectively), all of them are quite similar. They use a client-server or data-lake structure, where the data collected from the sensors is sent to a central repository (usually hosted in the cloud) for online or batch processing. These systems do not implement a cultivation context file (column 7) that, for example, allows the autonomous processes to self-adapt their services when the context changes. The only system that has such a capability is the proposed one, since it uses a blackboard architecture that keeps a record of both the cultivation context and the gardener context. 

Concerning the software services for the end-users (columns 8 to 12), most of them implement only monitors as the user profile, except the proposed system, which also supports gardeners. The monitors can review the current and historical data of the monitored variables, but cannot control the system as a gardener. Typically, the people use a web or mobile application to access these data (column 9). In the case of the systems reported in [9,40,47,55], this application is a COTS (commercial off-the-shelf) that is connected to the cloud to make use of the data recorded by the IoT system. Most of these applications deliver notifications and warnings to the end-users (column 10).

As mentioned before, the software support of these IoT solutions does implement a gardener context file that characterizes those users (column 11); therefore, the software has no chance of autonomously self-adapting the behavior of the services based on that information (column 12). This capability is provided only by the proposed system.

Concerning the data recorded and managed by the systems, most of these solutions keep an historical record (log file) with the values of the monitored variables (column 13). This log file does not include the record of the decisions made to control those variables, except for the last two systems shown in Table 11. Most of these systems access the current values of the monitored variables in batch (column 14), i.e., the mobile application accesses the log file or data lake to provide that information to the users, instead of asking the MCS for the current values in real-time.

## 8. Discussion 

In scenarios of voluntary adoption of technology, the potential adopters decide which technology seems to be more suitable, considering how well the technology features (e.g., functionality, usability and cost) align with the people’s needs and preferences [14,15]. Hydroponic cultivation at a family scale in urban settings requires the voluntary adoption of the support technology; in particular, at least a MCS and a software application that helps the gardener monitor and control the cultivation area. 

It is important to remark that the MCS and the software application are a combo designed to work together. Therefore, both components, individually and jointly, must be suitable for the gardener; otherwise, the solution has little chance of being adopted. 

Part of these needs and preferences come from the socio-economic and cultural context of the gardeners, e.g., income level, periods of free time, routines, language, and priorities to perform voluntary activities, including taking care of other people and the cultivation area. 

On the one hand, in the case of the MCS, low-cost, simple, extensible, and easy-to-deploy IoT systems tend to favor their adoption by Latin American urban gardeners. In particular, the monitoring and control system reported in this article accomplishes that purpose. First, the cost of the technology acquisition and operation is affordable for an important portion of the potential urban gardeners, and for crop-science researchers who can use the MCS in their experimental cultivations to study the gardening process or the gardeners. Moreover, it is simple, easy to deploy in a greenhouse, and can incrementally activate monitoring and control services depending on the sensors and actuators connected to the RPI-Case.

Nowadays, the improvements in MCS are focused mainly on optimizing the production of vegetables [7,8,44,47] or improving the technologies and mechanisms used in MCS implementations [32,33,36,37], but not on making these systems more adoptable for gardeners with particular requirements. The reasons behind these improvements are rooted in agriculture with commercial purposes, where the production process should be optimized, and the operation costs and human intervention tend to be minimized. Activities traditionally performed by people are now performed by electronic instruments, robots, and information and communication services.

Technology adoption in commercial agriculture is non-voluntary, and the farmers (usually employees of commercial firms) carry out the cultivation activities in conditions quite different to those of urban gardeners. These differences make most MCSs unsuitable (mainly, unaffordable) for this latter population, and call for the design of specific monitoring and control systems considering the capabilities and needs of these potential voluntary adopters, as shown in Table 11.

Concerning the software application that complements the MCS, the general scenario is similar to the previous one. However, the design aspects to address in the software product are different to those in the MCS, because, in this case, the application should consider the behavior of the gardeners and the human–computer interaction design aspects. For instance, its user interface should be in Spanish and perceived as usable. Moreover, it should support user profiles (roles) additional to the typical monitor, keep a record of the periods of workdays and the priorities of the involved people (i.e., a gardener context), and assume the cultivation process as a collaborative activity. Because the gardeners’ context is dynamic and there is no sensor capable of detecting these changes, it is required that the system behavior can be set on-demand (e.g., by the gardener or other person) to make the system aware of the context information and its evolution. As shown in Table 11, the proposed system is a step forward compared to the others reported in the related work.

Considering the analysis presented in the previous section, and to the best of our knowledge, this capability of the system to self-adapt its behavior based on the gardeners’ context is not present in the previous works reported in the literature. Therefore, we can say that the reported system represents a first step toward context-aware urban gardening; in this case, performed at a family scale and in a Latin American scenario. 

Additionally, the system also becomes an instrument to study the needs of certain clusters of urban gardeners, e.g., considering culture, age, or cultivation purposes. Based on that knowledge, technology designers can conceive IoT systems suitable for that target population or adjust existing ones with the same purpose. Next, we identify people who can benefit from using IoT-based context-aware systems for urban gardening:

− Urban gardeners can not only cultivate vegetables, but also tune the system behavior to make it fit with their temporary or permanent needs and preferences.
− Crop science researchers can perform the rapid prototyping and evaluation of vegetable-growing strategies by changing the parameters of the cultivation conditions, and thereby creating knowledge and recommendations for gardeners and technology designers.
− Technology designers and ICT researchers can evaluate the suitability of their IoT systems to support a specific end-user population. To do this, they can extend their solutions by implementing a log file of activities, the cultivation and gardener context, and a set of cultivation rules similar to those considered in the reported system. These are the inputs required to perform that assessment. Moreover, identifying the variables that are part of the gardeners’ context and understanding how to tune them will help designers create support systems suitable for particular end-user populations.
− Teachers can use the system to support various educational activities and promote urban gardening among younger populations, for instance, to show the students how to cultivate vegetables, and how the cultivation conditions influence the quality and quantity of the harvested products.

Urban gardening on a family scale is a context-aware activity, where two work contexts are involved, namely the cultivation context and the gardeners’ context. Both contexts influence not only the technology adoption but also the results of the cultivation process. Although previous research has made an initial step in considering some contextual variables (e.g., the scale and purpose of the activity), much more research and development is required. 

The state of the art shows IoT systems that can be configured to address cultivation methods, infrastructures, and products, but not to consider gardeners, as shown in Table 11. In this sense, the system and the experience reported in this article take a step forward, by identifying the end-users’ particularities as the key element not only to design technology for voluntary adoption scenarios, but also to envision the suitability of a certain technology for a target population. Therefore, this work opens a space to study gardening activities and design new IoT support systems for populations, for instance, older adults, teachers, students and gardeners from different cultures and socio-economic conditions. 

Finally, the capability of the system to dynamically adjust its behavior based on configurations allows for the rapid prototyping of cultivation strategies and discovering the preferences and needs of gardeners. This capability can support research activities in urban gardening from different perspectives, e.g., crop science, social science, computer science, and electronic engineering.

## 9. Conclusions and Future Work

As with any software system, fulfilling software requirements does not guarantee its adoption by end-users, especially when adoption is voluntary. Most IoT-based solutions that support urban gardening concentrate on the technical requirements of the system and are therefore commonly applied in contexts where end-users are not required to choose whether to adopt the technology; for instance, in commercial urban farming. In these cases, the user (typically an employee of a farming organization) must adjust their activities and preferences to use the system for cultivation tasks.

In contexts of voluntary technology adoption, such as in family-scale urban gardening, the situation differs. Gardeners often face constraints that affect how they perform cultivation, so supportive technology must account for these limitations to make the activity viable. Otherwise, such technology may not be adopted for these gardeners.

To begin exploring technology adoption for urban gardeners in Latin America, we used action research to develop, evaluate, and evolve an IoT system to support hydroponic cultivation. The system is capable of self-adapting its behavior, considering the cultivation and gardener’s contexts. This mechanism allows for tuning the system behavior by changing rules and configuration files, and thus considering the temporary or permanent needs and preferences of the gardeners. Therefore, it can be set to consider gardeners from different economic and socio-cultural conditions. The capability of the system to support the gardening activity and adjust its behavior during the cultivation process was evaluated through a tomato urban gardening experience conducted in Abancay, Peru. 

Before and during that experience, the system’s behavior was adjusted to meet the gardener’s needs and respond to dynamic cultivation conditions. This experience obtained positive results in terms of tomato production, but it principally demonstrated the system’s capability to self-adapt its behavior to consider the particularities of the urban gardener.

To the best of our knowledge, this is the first context-aware IoT system that self-adapts its behavior to address the gardeners’ context, and thus make the system more adoptable for these end-users. It represents a first step toward context-aware urban gardening; in this case, when the activity is performed at a family scale and in a Latin American scenario.

This article does not aim to resolve the specific challenges of urban gardening in Latin America, nor does it claim to prescribe how supportive technology should be designed for this demographic. Nonetheless, both the experience and the system show that it is feasible and beneficial to adapt system behavior to align with the needs of urban gardeners, often rooted in socio-cultural contexts. In this regard, within the scope of voluntary technology adoption, this article underscores the importance of designing system behavior not only with a socio-cultural perspective of the users, but also by providing mechanisms for easy adjustment. The system presented here demonstrates how this adaptability can be achieved.

The next step in this initiative involves improving the mechanisms to define and set context variables and rules, allowing the gardeners to perform these operations themselves using the mobile application. In addition, this experience will be replicated in other cities in Peru, with the support of the Peruvian government and international organizations, to continue exploring the relationship between the designing of IoT technology for urban gardening and the socio-cultural features of its adopters. 

## Figures and Tables

**Table 1:** Ideal ranges for tomato growth.

Variable	Acceptable Range
Temperature	15–28 °C
Humidity	60–85%
pH	5.5–6.5
EC	1.5–2.5 ms/cm

**Table 2:** Rest API and Telegram bot API.

API Command	Description
/start	Requests to authenticate the user and generate a user token.
/hum	Requests the value of the relative humidity in the greenhouse.
/temp	Requests the temperature value in the greenhouse (/cel: in Celsius, and /fa: in Fahrenheit).
/tempwater	Requests the temperature of the water for irrigation (/celwater: in Celsius, and /fawater: in Fahrenheit).
/ph	Requests the pH value of the nutrient solution.
/ec	Requests the electrical conductivity value of the nutrient solution.
/photo	Requests a photo of the cultivation area.
/video	Requests a video of the cultivation area (in two formats: /mp4 and /h264).
/bulb	Requests to turn on and off the light bulb: /onbulb and /offbulb.
/heater	Requests to turn on and off the heater: /onheater and /offheater.
/fan	Requests to turn on and off the fan: /onfan and /offfan.
/windows	Requests to turn on and off the windows: /onwindows and /offwindows.
/message	Allows for exchanging messages with other users of the greenhouse community: /send and /receive.
/help	Provides the list of endpoints available in the API.

**Table 3:** A summary of the Rest API.

API Command	Description
/user	It allows for creating, reading, updating and deleting users. It was designed to facilitate the management of users in a list, allowing them to update these data entities through CRUD operations.
/greenhouses	It allows for the creation, reading, updating, and deletion of greenhouses. It was designed to facilitate the management of greenhouses in a list, allowing them to update these data entities through CRUD operations.
/auth	It allows for registering and authenticating a user, and generating the user token.
/crop	It retrieves the information of a specific crop.
/actuators	It allows for creating, reading, updating and deleting actuators. This endpoint was designed to facilitate the management of actuators in a list.
/sensors	It allows for performing CRUD operations on the list of sensors available in a greenhouse.
/periods	It retrieves the information of crop periods.
/monitoring	It retrieves the monitoring information of the crop.
/notifications	It allows for reading, counting, and updating notifications.
/modules	It allows for the creation, reading, updating, and deletion of modules. It was designed to facilitate the management of modules in a greenhouse.
/help	It provides the list of endpoints available in the API.
…	…

**Table 4:** The data entities considered in the database.

Data Entity	Description
Greenhouse	Identifies the greenhouses and specify their main characteristics.
User	Identifies the registered users and the data required by the system to allow them to operate.
Roles	Specifies the roles that can be assigned to the user; only one role per greenhouse.
MC Rules	Establishes the rules to be considered by the monitor and controller components during the operation of the system. These rules can be set to consider the socio-cultural aspects of the gardeners.
MC Settings	Establishes the variables to be monitored and controlled during the operation, and their ranges of acceptable values.
Crops	Identifies the crops and their main characteristics and requirements.
Periods	Specifies the cultivation periods for every crop.
IoTDevices	Identifies the sensors and actuators deployed in each greenhouse.
LogFile	Establishes the sequence of actions, notifications, and events that happened in a particular greenhouse.
CultivData	For each variable monitored, it records the values of these variables during the whole cultivation period.

**Table 5:** Sensors and controllers.

Component	Description	Image
Raspberry Pi 4 Model B	A microcontroller that includes 4 GB of RAM (LPDDR), Dual-Band Wireless LAN 802.11 b/g/n/ac, Bluetooth 5.0, 2 USB 3.0 ports, 2 USB 2.0 ports, Gigabit Ethernet Power-over-Ethernet, 40 GPIO (General-Purpose Input/Output) connector pins, two micro-HDMI ports, a CSI camera port, a 3.5 mm combo jack for analog audio and composite video, a microSD card slot, and a USB-C connector.	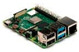
Temperature and Humidity sensor (DHT22)	The DHT22 sensor is a temperature and humidity sensor with features that bring it very close to high-precision ones. The output supplied is digital.	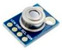
Pi Camera	Camera v2 for Raspberry Pi (8mpx, 1080P); it has a Sony IMX219 sensor.	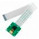
Tentacle for Raspberry Pi	A stackable add-on board for the Raspberry Pi to host EZO-class devices from Atlas Scientific to measure PH, electric conductivity (EC), and temperature.	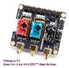
pH sensor	pH probe sensor + Circuit	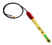
EC sensor	Electrical conductivity (EC) probe sensor + Circuit	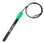
Temperature Sensor	Temperature sensor + Circuit	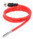

**Table 6:** Software programming languages and libraries.

Automation System:
*Python*, general-purpose programming language. *API-REST*, used to implement the Telegram bot. *Domoticz,* home automation system. *Firestore*, NoSQL database used to provide data persistence. *Refi App* is an open-source GUI tool to interact with Cloud Firestore.
**Web and Mobile Application:**
*Dart*, programming language used to implement the web and mobile applications. *JavaScript*, programming language and core technology of the Web. *NodeJs*, a runtime environment that allows for running JavaScript code on the server-side. *Flutter*, an open-source UI software development kit that is used to develop applications for Android, iOS, Linux, Mac, Windows, Google Fuchsia. It also allows developers to generate a Web version of an application. *API-REST*, used to implement TempHum. *Firestore* and *Refi App*, already explained in the previous section.

**Table 7:** CSUQ evaluation items.

Variable
**System Usefulness (Items 1–8):** 1. Overall, I am satisfied with how easy it is to use this system. 2. It was simple to use this system. 3. I could effectively complete the tasks and scenarios using this system. 4. I was able to complete the tasks and scenarios quickly using this system. 5. I felt comfortable using this system. 6. It was easy to learn to use this system. 7. I believe I could become productive quickly using this system. 8. The system gave error messages that clearly told me how to fix problems.
**Information Quality (Items 9–15):** 9. Whenever I made a mistake using the system, I could recover easily and quickly. 10. The information (such as online help, on-screen messages, and other documentation) provided with this system was clear. 11. It was easy to find the information I needed. 12. The information provided for the system was easy to understand. 13. The information was effective in helping me complete the tasks and scenarios. 14. The organization of information on the system screens was clear. 15. The interface of this system was pleasant.
**System Usability (Items 16–18):** 16. I liked using the interface of this system. 17. The system has all the functions and capabilities I expect it to have. 18. Overall, I am satisfied with this system.
19. Space for open comments

**Table 8:** Description of the tomato-growing periods.

Period	Description
Germination	This is the initial stage where the seed absorbs water and begins to sprout. It usually takes 5–10 days, depending on the variety and environmental conditions.
Seedling Stage	During this stage, the plant develops its first true leaves. This stage lasts for about 2–4 weeks, and the seedlings require proper care, including adequate watering and light.
Vegetative Growth	This stage focuses on leaf and stem development. This stage can last for 4–8 weeks, depending on the variety and growing conditions.
Flowering	Typically triggered by changes in daylength and temperature. Flowers begin to appear at the tips of the branches.
Fruit Development	After successful pollination, the flowers develop into fruits. This stage can take 4–6 weeks, depending on the variety and environmental conditions. Tomatoes grow, change color, and mature.
Ripening	The final stage is the ripening of the fruits, where they develop their final color and flavor. This process can take 1–2 weeks, and tomatoes are usually harvested when fully ripe.

**Table 9:** The tomato-growing periods in the greenhouse.

From	To	Period
1 September 2023	28 October 2023	(A) Germination
29 October 2023	17 November 2023	(B) Seedling Stage
18 November 2023	24 November 2023	(C) Vegetative Growth
25 November 2023	13 December 2023	(D) Flowering
14 December 2023	14 January 2024	(E) Fruit Development
15 January 2024	22 January 2024	(F) Ripening

**Table 10:** The production of tomatoes during the study.

Type	Total (kg)	Number of Plants	Average per Plant (kg)
Roma tomato	20.97	11	1.91
Globe tomato	20.85	7	2.98
Total	41.82	18	2.32

**Table 11:** An analysis of features of the hydroponic IoT systems to support urban gardening.

IoT System Report	Cultivation Scale	Monitored or Controlled Variables	Sensing and Controlling Mechanisms	IoT System Architect.	Software Architect.	System Self-Adapt. Using the Cultivation Context	Supported User Profiles	End-User App.	Message Delivery (Notif. and Warnings)	Gardener Context Record	System Self-Adapt. Using the Gardener Context	Log File of Activ.	Data Avail.
[4]	Family	Light intensity, humidity, temp., proximity	Only autonomous sensing	Layered uni-directional	Data lake	No	Monitor	Web app	Yes	No	No	Not Specified	Batch
[7]	Family and small cultivation	Humidity, EC, temp., pH, water flow/quality	Autonomous sensing and controlling	Layered uni-direct.	Not specified	No	No	Not available	No	No	No	No	Batch
[9]	Family and small cultivation	Humidity, temp., pH, water level/quality/flow, toxic gasses, light intensity	Autonomous sensing and controlling	Layered uni-directional	Data lake	No	Monitor	Web app (ThingSpeak)	Not Specified	No	No	Partial (historical record of variables)	Online
[39]	Family and small cultivation	Temp., humidity, EC, DO	Autonomous sensing and controlling	Master–slave	Client-server (UbiDot)	No	Monitor	Web app	Yes	No	No	Partial (historical record of variables)	Batch
[40]	Medium and large cultivation	Humidity, temp., water flow	Autonomous sensing and controlling	Layered uni-direct	Data lake	No	Monitor	Web app (ThingSpeak)	Not Specified	No	No	Partial (historical record of variables)	Online
[47]	Medium and large cultivation	Temp., humidity, vapor press. deficit	Autonomous sensing and controlling	Layered uni-direct	Data lake	No	Monitor	Mobile/web app (commercial)	Yes	No	No	Partial (historical record of variables)	Batch
[50]	Family and small cultivation	Humidity, temp., water level, NPK, LDR, capacitive soil moisture	Only autonomous sensing	Layered uni-direct	Client-Server	No	Monitor	Web and Mobile app	Yes	No	No	Partial (historical record of variables)	Batch
[51]	Family and small cultivation	Humidity, water temp., water/nutrient level, pH, EC	Only autonomous sensing	Layered uni-direct	Client-server	No	Monitor	Mobile app	No specified	No	No	Partial (historical record of variables)	Batch
[55]	Family and small cultivation	pH, EC, light intensity, humidity	Autonomous sensing and controlling	Layered uni-direct (IoT Antares)	Client-server	No	Monitor	Web and mobile app (IoT Antares)	No specified	No	No	Yes	Batch
Proposed IoT System	Family	Water temp., temp. humidity, pH, EC	Autonomous and on-demand sensing and controlling	Layered bi-directional	Backboard	Yes	Gardener and monitor	Web and mobile app	Yes	Yes	Yes	Yes	Online

## Data Availability

Data are contained within the article.
